# Effects of the Administration of *Bifidobacterium longum* subsp. *infantis* CECT 7210 and *Lactobacillus rhamnosus* HN001 and Their Synbiotic Combination With Galacto-Oligosaccharides Against Enterotoxigenic *Escherichia coli* F4 in an Early Weaned Piglet Model

**DOI:** 10.3389/fmicb.2021.642549

**Published:** 2021-04-16

**Authors:** Agustina Rodríguez-Sorrento, Lorena Castillejos, Paola López-Colom, Gloria Cifuentes-Orjuela, María Rodríguez-Palmero, José Antonio Moreno-Muñoz, Diana Luise, Paolo Trevisi, Susana María Martín-Orúe

**Affiliations:** ^1^Servicio de Nutrición y Bienestar Animal, Departament de Ciència Animal i dels Aliments, Universitat Autònoma de Barcelona, Bellaterra, Spain; ^2^Laboratorios Ordesa S.L., Parc Científic de Barcelona, Barcelona, Spain; ^3^Department of Agricultural and Food Science, University of Bologna, Bologna, Italy

**Keywords:** synbiotic, enterotoxigenic Escherichia coli, piglet, probiotic, prebiotic, galacto-oligosaccharides, *Bifidobacterium*, *Lactobacillus*

## Abstract

We evaluated the potential of multi-strain probiotic (*Bifidobacterium longum* subsp. *infantis* CECT 7210 and *Lactobacillus rhamnosus* HN001) with or without galacto-oligosaccharides against enterotoxigenic *Escherichia coli* (ETEC) F4 infection in post-weaning pigs. Ninety-six piglets were distributed into 32 pens assigned to five treatments: one non-challenged (CTR+) and four challenged: control diet (CTR−), with probiotics (>3 × 10^10^ CFU/kg body weight each, PRO), prebiotic (5%, PRE), or their combination (SYN). After 1 week, animals were orally inoculated with ETEC F4. Feed intake, weight, and clinical signs were recorded. On days 4 and 8 post-inoculation (PI), one animal per pen was euthanized and samples from blood, digesta, and tissues collected. Microbiological counts, ETEC F4 real-time PCR (qPCR) quantification, fermentation products, serum biomarkers, ileal histomorphometry, and genotype for mucin 4 (*MUC4*) polymorphism were determined. Animals in the PRO group had similar enterobacteria and coliform numbers to the CTR+ group, and the ETEC F4 prevalence, the number of mitotic cells at day 4 PI, and villus height at day 8 PI were between that observed in the CTR+ and CTR− groups. The PRO group exhibited reduced pig major acute-phase protein (Pig-MAP) levels on day 4 PI. The PRE diet group presented similar reductions in ETEC F4 and Pig-MAP, but there was no effect on microbial groups. The SYN group showed reduced fecal enterobacteria and coliform counts after the adaptation week but, after the inoculation, the SYN group showed lower performance and more animals with high ETEC F4 counts at day 8 PI. SYN treatment modified the colonic fermentation differently depending on the *MUC4* polymorphism. These results confirm the potential of the probiotic strains and the prebiotic to fight ETEC F4, but do not show any synergy when administered together, at least in this animal model.

## Introduction

Diarrhea is the second leading infectious cause of death – after pneumonia – among children younger than 5 years (Liu et al., 2015). The main pathogenic agents responsible for these diseases are viruses – namely rotavirus and calicivirus, which are responsible for 38 and 13% of the cases, respectively – followed by enteropathogenic and enterotoxigenic *Escherichia coli* (EPEC and ETEC, respectively), which contribute to 12 and 8%, respectively, of the deaths (Lanata et al., 2013). ETEC is also one of most common pathogens in pigs; it is responsible for post-weaning diarrhea syndrome (Luppi, 2017). ETEC carrying fimbriae F4 and F18 is considered as the most common pathogen and, thus, these pathogens are used to perform standardized challenges with *in vivo* trials (Luise et al., 2019a). Although *E. coli* is part of the normal intestinal microbiota, some strains, like the above mentioned, have developed pathogenic mechanisms to cause intestinal or extraintestinal disease (Clements et al., 2012). Due to the increasing development of antibiotic resistance in this organism (Poirel et al., 2018), new strategies for prevention and therapy must be implemented. Probiotics are defined by the Food and Agriculture Organization (FAO) as “live microorganisms which, when administered in adequate amounts, confer a health benefit on the host.” These advantageous effects are strain dependent and can include gastrointestinal disorder prophylaxis and treatment, immune system enhancement, cancer prevention, and cholesterol normalization, among others (Kechagia et al., 2013). Regarding the potential of probiotics to ameliorate diarrhea caused by ETEC, different strains of bifidobacteria and lactobacilli have proven antagonistic activity against *E. coli in vitro* (Vazquez-Gutierrez et al., 2016; Fijan et al., 2018; Song et al., 2019). There have also been positive results when tested *in vivo* (Romond et al., 1997; Kumar et al., 2016). Our previous work has shown that the particular strain *Bifidobacterium longum* subsp. *infantis* CECT 7210 can reduce ileal colonization by ETEC, a phenomenon that improves the local immune response in a piglet model (Barba-Vidal et al., 2017). Regarding lactobacilli strains, *Lactobacillus rhamnosus* has been reported to have positive effects against pathogenic *E. coli*, although the benefits could depend on the probiotic strain. In this sense, *L. rhamnosus* HN001 has been demonstrated to reduce enterohemorrhagic *E. coli* translocation and to increase immunoglobulin A (IgA) concentration and blood leukocyte phagocytic activity in mice (Shu and Gill, 2001). However, administration of *L. rhamnosus* ATCC 53103 to post-weaning piglet infected with ETEC F4 does not confer protection (Trevisi et al., 2009). A combination of *B. longum* and *L. rhamnosus* HN001 has also been reported as an effective way to modulate the intestinal environment in humans, with the reductions of potential harmful bacteria and an increase of beneficial ones (Toscano et al., 2017).

On the other hand, prebiotics can also be useful against pathogenic bacteria (de Vrese and Marteau, 2007). Galacto-oligosaccharides (GOSs) are obtained from lactose by transgalactosylation reactions catalyzed by β-galactosidases. These reactions result in a chain of galactose units with a terminal glucose unit (Tzortzis, 2009). This prebiotic has been associated with increases in lactobacilli and bifidobacteria *in vitro* as well as in human clinical trials (Grimaldi et al., 2016; Canfora et al., 2017; Paganini et al., 2017). Moreover, GOS are considered to be highly similar to oligosaccharides from human milk (HMO), a factor that makes it an attractive prebiotic when designing synbiotics with *B. longum* subsp. *infantis* or other probiotic strains isolated from the infant intestine. Notably, genomic adaptations for HMO utilization have been described for *B. longum* subsp. *infantis* (Sela et al., 2008). Furthermore, GOS have been shown to interfere with *E. coli* adhesion to tissue culture cells (Shoaf et al., 2006). Moreover, recent studies have demonstrated the ability of the probiotic strain *B. longum* subsp. *infantis* CECT 7210 to metabolize GOS as substrate, promoting a higher growth than the other prebiotics. This combination has also shown significant antimicrobial properties against *E. coli* (Ruiz et al., 2020).

Based on this information, we hypothesized that the efficacy of combined probiotic strains of *Bifidobacterium* and *Lactobacillus* will be increased by the addition of fermentable carbohydrates that may promote their growth and activity in the gut. Hence, the objective of this work was to evaluate whether combining multi-strain probiotic, composed of *B. longum* subsp. *infantis* CECT 7210 and *L. rhamnosus* HN001, with GOS could improve activity against ETEC F4 in post-weaning piglets.

## Materials and Methods

The trial was performed at the Experimental Unit of the Universitat Autònoma de Barcelona (UAB) and received prior approval (Permit No. CEEAH: 4026 DMAH: 10118) from the Animal and Human Experimental Ethical Committee of this Institution. The treatment, management, housing, husbandry, and slaughtering conditions conformed to European Union Guidelines (Directive 2010/63/EU; European Commission, 2010). All efforts were made to minimize animal suffering.

### Animals, Housing, and Experimental Design

This trial was carried out as level 2 high-risk biosecurity procedures and involved personnel who had received appropriate training. A total of 96 male piglets (Landrace × Large White) × Pietrain, 21 (±2) days of age and weighting 5.04 ± 0.32 kg, were used. Only males were selected to reduce possible residual variability associated to sex as sample size is limited in trials involving pathogen challenges. All animals came from a high-sanitary-status farm in which mothers were not vaccinated against *E. coli*.

Piglets were transferred to the experimental unit located in the UAB, which consisted of four boxes of eight pens each (32 pens, three animals per pen). Each 2 m^2^ pen comprised a feeder and water nipple to provide feed and water *ad libitum*. All weaning rooms were equipped with an automatic heater and forced ventilation, and each pen had an individual heating light.

At arrival, animals were distributed among treatment groups according to their initial body weight. The trial was a completely randomized design that included five experimental groups: (i) positive control (CTR+), pigs not challenged with ETEC but orally inoculated with a sterile placebo solution; (ii) negative control (CTR−), pigs orally challenged with ETEC F4; (iii) probiotic (PRO), pigs receiving a combination of *B. longum* subsp. *infantis* CECT 7210 and *L. rhamnosus* HN001 and orally dosed with ETEC F4; (iv) prebiotic (PRE), pigs receiving galacto-oligosaccharides and orally dosed with ETEC F4; and (v) synbiotic (SYN), pigs receiving the combination of the products supplied in the PRO and PRE groups and orally dosed with ETEC F4. These challenged groups were equally distributed in three of the four rooms, while one full room was kept for non-inoculated control pigs; hence, the design was unbalanced. Each experimental group had six replicates, except for the non-challenged groups, which had eight replicates. In the challenged rooms, probiotic, and synbiotic treatments were distributed within four pens on one side of the room, and the control and prebiotic pens were on the other side of the room, separated by a corridor in between to avoid cross-contamination.

### Probiotic Strains, Prebiotic, and Diets

The tested probiotics were *B. longum* subsp. *infantis* CECT 7210, supplied by Laboratorios Ordesa S.L., and *L.s rhamnosus* HN001 (Danisco USA Inc.). Both strains were provided lyophilized, containing 5 × 10^10^ and 3 × 10^10^ colony forming units (CFU) per gram of product, respectively, in a maltodextrin base. The lyophilized probiotics were mixed daily into the feed for a final dosage of 5.5 × 10^7^ and 3.3 × 10^7^ CFU/g, respectively. The feed was totally replaced daily. Before the trial, we confirmed the viability of the probiotics in the dry feed during the day.

The experimental prebiotic containing galacto-oligosaccharides (GOS) was in a syrup form and was thoroughly mixed with the diet by hand every day to guarantee a homogenous blend (no visual lumps). The final GOS concentration was 5% (w/w) trying to reach the amount of oligosaccharides provided by human milk (5–10 g/L; Kunz et al., 2017) and also based in previous studies supplementing GOS to neonatal and weaned piglets (Alizadeh et al., 2016; Tian et al., 2019; Wang et al., 2019). When mixed in the SYN diet, GOS was mixed prior to the probiotics.

Pre-starter diets were formulated in concordance with the nutrient requirement standards for pigs [National Research Council (NRC), 2012] and given in a mash form. The possible amino acid dilution in the PRE and SYN diets due to the incorporation of the prebiotic was compensated by the addition of synthetic amino acids: 0.5 g L-valine, 0.9 g L-lysine HCl, 1.2 g DL-methionine, 0.5 g L-threonine, and 0.2 g L-tryptophan per kg of feed. Details for their ingredient and chemical composition are given in Table 1.

**Table 1:** Ingredient and nutritional composition of the diets.

Ingredients (g/kg FM)	CTR/PRO	PRE/SYN
Maize	207.4	196.3
Wheat	180.0	170.0
Barley 2 row	170.0	160.9
Extruded soybean	149.1	141.1
Sweet whey-powder (cattle)	100.0	94.6
Fish meal	60.0	56.8
Soybean meal 44	80.0	75.7
Whey-powder 50% fat	25.0	23.6
Mono-calcium phosphate	6.8	6.4
Calcium carbonate (CaCO_3_)	3.9	3.6
L-Lysine HCL	4.5	5.0
Vit-Min Premix^*^	4.0	3.7
Sodium chloride (marine salt)	2.5	2.3
DL-Methionine 99	2.6	3.6
L-Threonine	2.3	2.6
L-Tryptophan	0.6	0.7
L-Valine	1.5	1.9
Prebiotic	0	5
**Analyzed composition (g/kg FM)**	**CTR/PRO**	**PRE/SYN**
Dry matter	920.2	916.1
Ashes	49.6	48.2
Crude fat	64.1	61.6
Crude protein	203.2	191.1
Neutral detergent fiber	92.7	91.0
Acid detergent fiber	32.7	32.4
**Calculated composition (g/kg FM except ME)**		
Metabolizable energy (kcal/kg FM)	3,378	3,213
Crude protein	214	205
Lysine	15	15
Methionine	6	7
Threonine	10	10
Tryptophan	3	3
Valine	11	10
Cysteine	3	3
Leucine	15	15
Isoleucine	9	8

*indicates that values of p are <0.05 and statistical difference is present.

### *Escherichia coli* F4 Strain

The bacterial strain of ETEC F4 used was isolated from feces of 14-week old pigs and provided by the Infectious Diseases Laboratory (Ref. 30/14) of the UAB. This strain presented the following virulence factors: F4ab, F4ac, LT, STb, and EAST1, and it was negative for K99, F6, F18, F41, STa, VT1, VT2, and EAE. The oral inoculum was prepared by a 12-h overnight incubation at 37°C in brain heart infusion broth (Oxoid; Hampshire, England) with slow agitation (250 rpm) in an orbital incubator. A total volume of 6 ml from the culture was given directly to the animals; the final concentration was 1 × 10^9^ CFU/ml. Inoculum concentrations were also determined before the inoculation by McFarland standards and were plated in Luria Agar (LA; made in house: tryptase, yeast extract, NaCl, and agar, Oxoid; Hampshire, United Kingdom) for the same day manual plate counting.

### Experimental Procedure

The experiment lasted 15 days. Animals were offered the experimental diets from the first moment after arrival. After an adaptation period of 7 days (day 7), animals were orally challenged with the pathogen. The inoculum was given to the challenged groups *via* the oral route as a single dose of 6 ml of ETEC F4 bacterial culture containing 6 × 10^9^ CFU. The same amount of sterile broth was administered to non-challenged piglets. To ensure that the animals had a full stomach at the moment of the oral challenge, pigs were starved for 12 h (night period from 8:00 pm to 8:00 am) and feed was reintroduced 30 min before the inoculation.

From the challenge onward, animals were checked daily to determine clinical signs and evaluate their post-inoculation status – always by the same person. The fecal score was measured using a scale: 1 = solid and cloddy; 2 = soft with shape; 3 = very soft or viscous liquid; and 4 = watery or with blood. The rectal temperature was assessed with a digital thermometer (Accuvet, Sanchung City, Taiwan) on days 1 and 2 PI.

For microbiological analysis, fecal samples were collected aseptically after spontaneous defecation or by digital stimulation at arrival and on the day of the inoculation (day 0 PI). Fecal samples were always obtained from the largest animal of each pen (*N* = 32), except at day 4 PI, when the average piglet was sampled.

Animal performance was monitored. Individual body weight was registered at arrival (day 7) and on days 0, 4, and 8 PI, and feed intake was monitored daily, associated with the regular feed change to maintain probiotic viability. The average daily gain (ADG) and average daily feed intake (ADFI) were calculated by pen for the adaptation period (days 0–6), days 0–4 PI (days 7–11), and days 4–8 PI (days 12–15). The G:F ratio was calculated by pen for the adaptation period (days 0–6), post inoculation period (days 7–15), and total length of the trial (days 0–15). The animals received no antibiotic treatment.

On days 4 and 8 PI, one pig per pen was euthanized. On day 4 PI, the selected animal was the one with an intermediate initial BW, while on day 8 PI, the chosen piglet was the heaviest from each pen. Animals were euthanized and sequentially sampled during the morning of each day (between 8:00 and 13:00 h). An intramuscular injection containing 20 mg/kg ketamine (Ketamidor; Wels, Austria) and 2 mg/kg of xylazine (Xilagesic; Les Franqueses del Vallès, Spain) was given to the animals to induce deep sedation. Prior to injection of the euthanasia drug, 10 ml sample blood was taken from each animal *via* venipuncture of the cranial cava vein using 10 ml blood collection tubes without anticoagulant (Aquisel; Madrid, Spain). Right after blood sampling, pigs were euthanized with an intravenous injection of sodium pentobarbital (140 mg/kg BW, Euthasol, Le Vet B.V., Oudewater, Netherlands). Once dead, animals were bled, the abdomen was immediately open, and the gastrointestinal tract extracted.

A fecal sample taken from rectum was kept in ice and used for culture-based microbiology; it was analyzed within 4 h of collection. Subsequently, content from the ileum and proximal colon was collected and homogenized prior to pH determination with a pH meter calibrated on each day of use (Crison 52–32 electrode, Net Interlab; Barcelona, Spain). Subsamples of colonic and ileal contents were collected for different analyses. One aliquot of colonic content was kept frozen at −80°C for ETEC F4 quantification by real-time PCR (qPCR). A set of ileal and colonic digesta aliquots were stored at −20°C in H_2_SO_4_ solution (3 ml of content plus 3 ml of 0.2 N H_2_SO_4_) for ammonia (NH_3_) determination and an additional sample (~10 g) was also frozen at −20°C until analysis for short-chain fatty acids (SCFA) and lactic acid determination.

From each animal, 5 cm of distal ileum were collected, washed thoroughly with sterile phosphate-buffered saline (PBS), opened longitudinally, and scraped with a glass microscope slide to obtain the mucosa sample to determine the number of enterobacteria and coliforms attached to the intestinal mucosa. A subsample was also stored at −80°C for ETEC F4 quantification by qPCR.

For the histological study, 1 cm of ileum was removed, opened longitudinally, and washed thoroughly with 4% formaldehyde solution (Panreac; Castellar del Vallès, Spain) before fixing them by immersion in the same solution. Blood samples were centrifuged (3,000 *g* for 15 min) after blood coagulation; the obtained serum was stored at −20°C.

### Analytical Procedures

#### Feed Analysis

Chemical analyses of the diets, including dry matter (DM), ash, crude protein, and diethyl ether extract, were performed according to the Association of Official Agricultural Chemists standard procedures (AOAC International, 1995). Neutral detergent fiber and acid-detergent fiber were determined according to the method of Van Soest et al. (1991).

#### Microbiological Analysis

For enterobacteria and coliform counts, samples were serially diluted in Ringer’s lactate solution (Sigma-Aldrich, Madrid, Spain) and proper dilutions seeded in MacConkey agar (Oxoid; Madrid, Spain) and eosin methylene blue agar (Scharlab; Barcelona, Spain). Plaques were incubated for 24 h at 37°C and colonies were manually counted. From colon content and ileal mucosal scrapings, the total bacterial DNA was extracted using QIAmp DNA Stool Mini Kit (Qiagen; West Sussex, United Kingdom) following the manufacturer’s instructions. The ETEC F4 concentration was than assessed by qPCR targeting the gene coding the F4 fimbria of *E. coli*, according to the procedure described by Hermes et al. (2013), using SYBR green dye with the ABI 7900 HT Sequence Detection System (PE Biosystems, Warrington, United Kingdom) with optical grade 96-well plates. The results were scored in five levels according to the number of gene copies per gram of fresh matter (FM). Scores were defined as following: negative = less than 4 logarithmic units of gene copies per gram FM; low = 4–5.5 logarithmic units of gene copies per gram FM; medium = 5.5–7 logarithmic units of gene copies per gram FM; high = 7–8.5 logarithmic units of gene copies per gram FM; and very high = more than 8.5 logarithmic units of gene copies per gram FM.

#### Short-Chain Fatty Acids, Lactic Acid, and Ammonia Analyses

Short-chain fatty acids and lactic acid analyses were performed on ileal and colonic digesta samples by gas liquid chromatography. Samples were subjected to acid-base treatment prior an ether extraction and derivatization with N-(tertbutyldimethylsilyl)-N-methyl-trifluoroacetamide (MBTSTFA) plus 1% tert-butyldimethylchlorosilane (TBDMCS) agent, using the method of Richardson et al. (1989), modified by Jensen et al. (1995).

Ammonia concentrations were assessed on ileal and colonic digesta samples using a gas-sensitive electrode (Hatch Co.; CO, United States) combined with a digital voltmeter (Crison GLP 22, Crison Instruments, S.A.; Barcelona, Spain) and following a procedure described by Hermes et al. (2009). Samples were diluted (1:2) in 0.16 M NaOH and, after homogenization, were centrifuged at 1500 *g* for 10 min. Once the ammonia was released, it was measured in the supernatants as a change in voltage (in mV).

#### Serum Analysis

Serum samples were analyzed for Tumor Necrosis Factor-α (TNF-α) and pig major acute-phase protein (Pig-MAP). The concentrations of TNF-α were determined by enzyme-linked immunosorbent assay (ELISA) using the Quantikine Porcine TNF-α kit (R&D Systems; Minneapolis, United States), using a 4-parameter logistic regression (4PL) fit for calibration curve adjustment. Pig-MAP concentrations were determined by a turbidimetric method using the TURBOVET Pig-MAP (Acuvet Biotech; Zaragoza, Spain). Samples were not diluted in any of the analyses.

#### Histological Analysis

For histological study, ileal samples were dehydrated and embedded in paraffin wax, sectioned at 4 μm, and stained with hematoxylin and eosin. Measurements of 10 different villus-crypt complexes per sample were considered including counting of intraepithelial lymphocytes (IEL), Goblet cells (GC), and the number of mitotic cells in each complex. Analyses were performed with a light microscope (BHS, Olympus, Barcelona, Spain) following the procedure described by Nofrarías et al. (2006).

#### Piglet Genotyping for ETEC Susceptibility

The mucin 4 (*MUC4*) gene has been suggested as one of the candidate gene associated with piglet susceptibility to ETEC F4 (Jørgensen et al., 2003; Luise et al., 2019a). Thus, in the present study, the animals were genotyped for the polymorphism described by Jørgensen et al. (2003). For *MUC4* genotype determination, hair follicles were collected from 81 pigs. DNA was extracted following the procedure described by Luise et al. (2019b). A restriction fragment length polymorphism PCR (PCR-RFLP) was performed following the guidelines described by Jørgensen et al. (2003). Pigs were classified into two groups: susceptible pigs having the *MUC4*^GG^ or *MUC4*^CG^ genotype (*MUC4*+) or resistant pigs having *MUC4*^CC^ homozygotes (*MUC4*−).

### Statistical Analysis

The results are expressed as means with their standard errors unless otherwise stated. Microbiological counts were log transformed for analysis. A two-way analysis of variance (ANOVA) was used to examine the effect of the five experimental treatments and the *MUC4* (*MUC4*+ and *MUC4*−) with the following model:

$$\mathrm{Yijk}=m+{\mathrm{treat}}_{i}+{\mathrm{MUC4}}_{j}+\left(\mathrm{treat}*{\mathrm{MUC4}}_{\mathrm{ij}}\right)+e_{\mathrm{ij}},$$

where Y_ijk_ relates to each observation of the outcome variable, m is the global mean, treat_i_ is the main effect of treatment, MUC4_j_ is the main effect of *MUC4* polymorphism, and treat*MUC4_ij_ corresponds to the interaction between treatment and *MUC4*. Finally, e_ij_ is the experimental error term. Regarding *MUC4* effect and interaction term, they were removed from the model when found not to be significant.

The effects on the post-slaughter measurements were examined using the R v3.4 (R Core Team, 2013) lm function for two-way ANOVA, with the treatment and *MUC4* effects as main effects. When the *MUC4* effect was not observed (*p* > 0.05), one-way ANOVA was performed with only the treatment effect. For *E. coli* F4 prevalence values, data were subjected to frequency analysis using the fisher.test function in the same package.

ADFI and daily fecal scores were also analyzed using the lme4 package (Bates et al., 2015) lmer function for a generalized linear mixed model (GLMM) with a treatment-by-time interaction term and considering the animal as the random effect.

The experimental unit was the pen. For all analyses, significance was set at *p* < 0.05 and *p* ≥ 0.05, but ≤ 0.10 were considered to indicate a statistical trend. When treatment effects were established, the mean comparison was adjusted with the Tukey-Kramer test. Data are presented as means and residual standard error (RSE).

## Results

Following the oral challenge with ETEC, animals developed moderate clinical signs of diarrhea that began to resolve spontaneously at the end of the study. Eight spontaneous casualties occurred (by group: 3 CTR−, 3 PRE, 1 PRO, and 1 SYN), and no humane euthanasia was required. Table 2 presents the analysis of mucin 4 (*MUC4*) polymorphism for each treatment group.

**Table 2:** Distribution of resistant and susceptible animals for *MUC4* gene in each experimental group.

Treatment	*MUC4* genotype	
	Resistant	Susceptible
**Total animals**		
CTR+	14	6
PRO	10	7
PRE	8	7
SYN	7	10
CTR−	7	5
***p* = 0.529**		
**Animals sampled at day 4 PI**		
CTR+	4	4
PRO	5	1
PRE	3	3
SYN	1	5
CTR−	3	3
***p* = 0.276**		
**Animals sampled at day 8 PI**		
CTR+	7	1
PRO	2	4
PRE	3	3
SYN	4	2
CTR−	4	2
***p* = 0.294**		

CTR+: non-inoculated animals receiving placebo; PRO: inoculated animals receiving the probiotics; PRE: inoculated animals receiving the prebiotic; SYN: inoculated animals receiving the synbiotic; CTR−: Inoculated animals receiving placebo. *N* = 6 for all groups except for non-challenged animals (*N* = 8). p values were obtained using Fisher’s exact test in R software. *MUC4*, mucin 4; PI, post-inoculation.

### Performance Parameters

Table 3 presents the results obtained for body weight (BW), average daily feed intake (ADFI), average daily weight gain (ADG), and gain to feed (G:F) ratio. During the 1st week, the animals from all experimental groups had a similar feed intake. However, after pathogen inoculation, all challenged groups showed numerical reductions in intake, although the differences only reached statistical significance for the CTR− and SYN groups for the days 0–4 post-inoculation (PI) period (*p* = 0.002) and for the SYN group for the days 4–8 PI period (*p* < 0.001).

**Table 3:** Effects of experimental treatments on feed intake and weight gain.

	Treatments					RSD	*p*
	CTR+	PRO	PRE	SYN	CTR−		
**Body weight (kg)**							
Initial	5.02	5.09	5.01	5.05	5.03	*0.142*	*0.880*
Final	7.24	6.89	7.25	6.48	6.58	*0.726*	*0.223*
**Average daily feed intake (g/day)**							
Adaptation	105.1	111.8	118.8	102.3	83.9	*27.86*	*0.285*
0–4 PI	213.0^a^	185.3^ab^	179.0^ab^	124.1^b^	130.1^b^	*42.35*	0.002^*^
4–8 PI	404.8^a^	350.5^a^	330.9^ab^	207.7^b^	306.2^ab^	*70.83*	<0.001^*^
**Average daily gain (g/day)**							
Adaptation	40.7	46.2	64.6	52.0	34.4	*38.93*	*0.707*
0–4 PI	155.4^a^	74.2^ab^	26.7^b^	−13.7^b^	−44.7^b^	*71.50*	<0.001^*^
4–8 PI	317.6^a^	329.3^a^	333.0^a^	198.5^b^	282.5^b^	*82.15*	0.047^*^
**Gain:Feed ratio**							
Adaptation	0.34	0.04	0.36	0.54	0.50	*0.665*	*0.727*
PI	0.76	0.52	0.75	0.54	0.44	*0.328*	*0.300*
Total	0.68	0.50	0.65	0.59	0.51	*0.185*	*0.298*

CTR+: non-inoculated animals receiving placebo; PRO: inoculated animals receiving the probiotics; PRE: inoculated animals receiving the prebiotic; SYN: inoculated animals receiving the synbiotic; CTR−: Inoculated animals receiving placebo. *N* = 6 for all groups except for non-challenged animals (*N* = 8). Values of *p* were obtained by an ANOVA using the generalized linear procedure in R software. ^a,b^Indicate statistically significant differences between groups. PI, post-inoculation; RSD, relative standard deviation. To differentiate RSD and values of p from the results numbers, they are in italics. *indicates that values of p are <0.05 and statistical difference is present.

**Figure 1:**
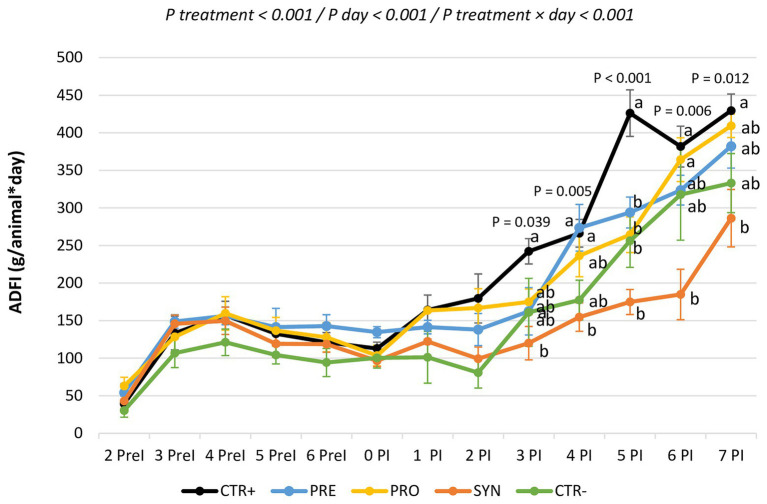
Evolution of feed consumption for the experimental groups during the entire experimental period. CTR+: non-inoculated animals receiving placebo; PRO: inoculated animals receiving the probiotics; PRE: inoculated animals receiving the prebiotic; SYN−: inoculated animals receiving the synbiotic; CTR−: inoculated animals receiving placebo. *N* = 6 for all groups except for non-challenged animals (*N* = 8). a and b indicate statistically significant differences between groups. Bars correspond to the standard error. ADFI, average daily feed intake; PreI, pre-inoculation; PI, post-inoculation.

Figure 1 shows the evolution of feed intake per day during the experimental period. There were statistical differences between groups beginning on day 3. The SYN group was the most affected: this group consistently showed the lowest feed intakes; being significantly different from CTR+ on days 3, 4, 5, 7, and 8 PI.

Similarly, ADG did not differ between treatments during the 1st week of the trial, but it decreased after the challenge. During the first period after the inoculation (days 0–4 PI), all the challenged groups showed significant decreases in growth, except for the PRO group, in which numerical reductions in weight gains did not reach statistical significance. In the second phase (days 4–8 PI), the PRO and PRE groups showed a fast recovery of weight gain. Indeed, they reached similar levels (even numerically higher) to non-inoculated piglets. However, the SYN group showed a tendency toward lower weight gain compared to the CTR+ group (*p* = 0.083). There were no differences among the groups for G:F ratio during the trial.

### Clinical Signs

**Figure 2:**
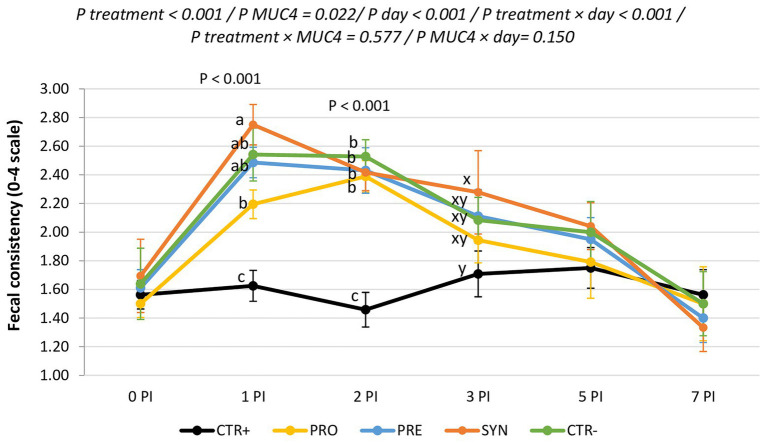
Evolution of average fecal scores for the different experimental groups during the post-inoculation (PI) period. CTR+: non-inoculated animals receiving placebo; PRO−: inoculated animals receiving the probiotics; PRE−: inoculated animals receiving the prebiotic; SYN−: inoculated animals receiving the synbiotic; CTR−: inoculated animals receiving placebo. *N* = 6 for all groups except for non-challenged animals (*N* = 8). *MUC4* represents the effect of polymorphism of the *MUC4* gene. a,b, and c indicate statistically significant differences between groups. x and y indicate statistical trends among groups. Bars correspond to the standard error. PI, post-inoculation.

Figure 2 shows the evolution of fecal consistency along with the post-infection period. ETEC F4-challenged animals showed higher fecal scores (lower fecal consistency) immediately after the inoculation; these differences were significant on days 1 and 2 PI and recovered from day 3 PI onward. *MUC4*+ animals had worse scale numbers compared to *MUC4*− animals on days 1 and 2 PI (2.03 vs. 2.47; *p* = 0.009 and 1.95 vs. 2.34; *p* = 0.013 for not susceptible and susceptible on days 1 and 2 PI, respectively). Among the challenged groups, the PRO group had the best consistency and the SYN group the worst (1.60, 1.87, 1.95, 2.07, and 2.10 for CTR+, PRO, PRE, SYN, and CTR−, respectively). There was no treatment × *MUC4* interaction. Rectal temperature was within the normal range and was not affected by the challenge, experimental diets, or *MUC4* status.

### Microbiological Analysis

Table 4 shows enterobacteria and coliform plate counts from fecal samples taken at the arrival of the animals, before the oral challenge, and at days 4 and 8 PI. The table also includes plate counts from ileal mucosa scrapings at day 4 and 8 PI.

**Table 4:** Effects of experimental treatments on enterobacteria and coliform counts in fecal samples and ileal scrapings.

	Treatments					RSD	*p*
	CTR+	PRO	PRE	SYN	CTR−		
**Enterobacteria (log CFU/g MF)**							
**Feces**							
Arrival	9.35	9.76	8.10	8.91	7.87	1.259	*0.242*
Day 0 PI	8.82^ab^	10.93^a^	10.76^a^	7.55^b^	10.12^ab^	1.685	0.001^*^
Day 4 PI	6.41	6.63	6.92	6.29	8.52	1.501	0.043^*^
Day 8 PI	5.40^b^	5.34^b^	6.85^a^	5.79^b^	6.75^a^	0.441	<0.001^*^
**Ileal scrapings**							
Day 4 PI	7.59	7.61	7.54	7.46	7.41	*0.201*	0.393
Day 8 PI	6.62^b^	6.62^b^	7.62^a^	7.59^a^	7.24^ab^	*0.423*	<0.001^*^
**Total coliforms (log CFU/g FM)**							
**Feces**							
Arrival	9.18	9.70	8.09	8.81	7.87	1.325	*0.335*
Day 0 PI	8.34^b^	10.83^a^	10.66^a^	5.86^b^	9.72^ab^	1.248	<0.001^*^
Day 4 PI	6.35^ab^	6.31^ab^	6.82^ab^	5.90^b^	8.48^a^	1.380	0.008^*^
Day 8 PI	5.31^b^	5.22^b^	6.83^a^	5.71^b^	6.62^a^	0.441	<0.001^*^
**Ileal scrapings**							
Day 4 PI	7.48	7.46	7.46	7.42	7.31	*0.222*	*0.642*
Day 8 PI	5.90^c^	6.55^bc^	7.18^ab^	7.65^a^	7.29^ab^	*0.576*	<0.001^*^

CTR+: non-inoculated animals receiving placebo; PRO: inoculated animals receiving the probiotics; PRE: inoculated animals receiving the prebiotic; SYN: inoculated animals receiving the synbiotic; CTR−: inoculated animals receiving placebo. *N* = 6 for all groups except for non-challenged animals (*N* = 8). Values of *p were* obtained by an ANOVA using the generalized linear procedure in R software. ^a,b^Indicate statistically significant differences between groups. CFU, colony forming units; PI, post-inoculation; RSD, relative standard deviation. To differentiate RSD and values of p from the results numbers, they are in italics. *indicates that values of p are <0.05 and statistical difference is present.

There were no differences between experimental groups on the day of arrival. However, at the end of the adaptation phase, the SYN group showed lower values of fecal enterobacteria (*p* = 0.001) and coliforms (*p* < 0.001) compared to the PRE and PRO groups. On day 4 PI, the SYN group was the only one with lower fecal coliforms counts compared to the CTR− group (*p* = 0.023). On day 8 PI, only the PRE and CTR groups maintained higher plate counts compared to non-challenged pigs (*p* < 0.001 for both groups).

*MUC4* also had an impact on fecal counts of enterobacteria and coliforms on day 4 PI: resistant animals had lower counts compared to susceptible carriers (enterobacteria 6.27 vs. 7.44 CFU/g; P *MUC4* = 0.015; coliforms 6.23 vs. 7.22 CFU/g; P *MUC4* = 0.010). The interaction between *MUC4* and treatment was not significant, except for fecal enterobacteria before the challenge, when there were differences in the SYN group compared to the other treatment groups only in non-susceptible pigs who had a lower number of enterobacteria (9.17, 10.86, 10.77, 5.92, and 10.05 CGU/g for CTR+, PRO, PRE, SYN, and CTR−, respectively; P interaction = 0.003).

Regarding ileal scrapings, only on day 8 PI were there differences between treatments (*p* < 0.001). Specifically, the PRO group was the only one not that different from the CTR+ group.

**Figure 3:**
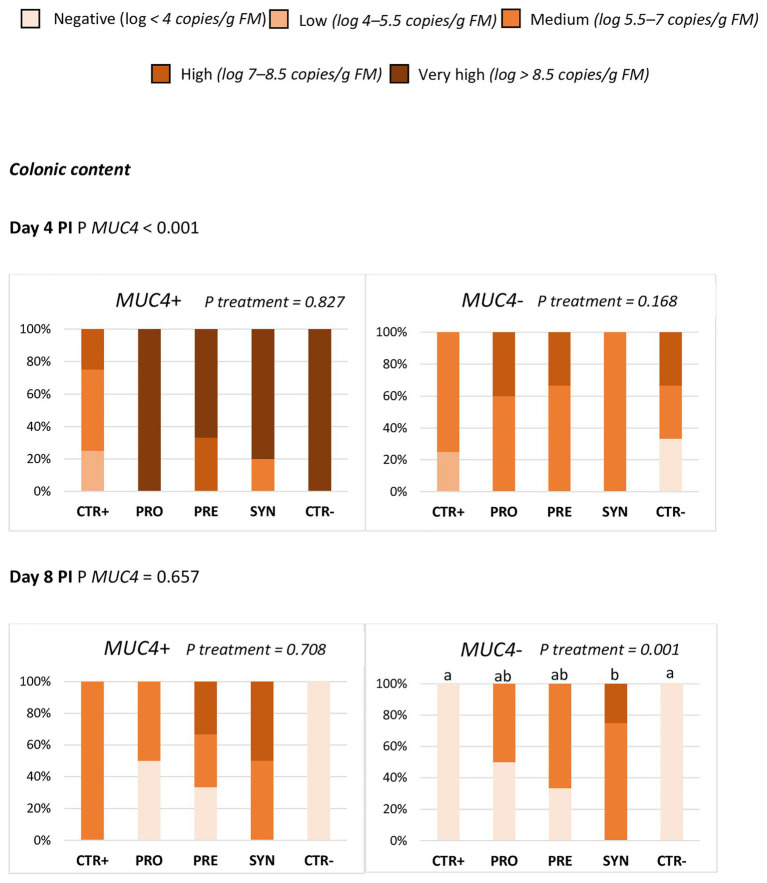
Effect of *MUC4* polymorphism on the percentage of animals with different levels of ETEC F4 counts in colon content on days 4 and 8 post-inoculation (PI). Different animals were sampled on days 4 and 8 post-inoculation (PI). CTR+: non-inoculated animals receiving placebo; PRO−: inoculated animals receiving the probiotics; PRE: inoculated animals receiving the prebiotic; SYN: inoculated animals receiving the synbiotic; CTR−: inoculated animals receiving placebo. *N* = 6 for all groups except for non-challenged animals (*N* = 8). Values of *p* were obtained using Fisher’s exact test in R software. a and b indicate statistically significant differences between groups. *MUC4*+, Mucin 4 susceptible genotype; *MUC4*−, Mucin 4 resistant genotype; PI, post-inoculation.

The ETEC F4 quantification results by qPCR in colonic digesta and ileal scrapings showed that *MUC4* status significantly affected the prevalence of ETEC F4 in colonic digesta at day 4 PI. The challenge effect was much clearer in carrier animals (P *MUC4* < 0.001), with no significant difference among treatments (Figure 3). At day 8 PI, there was an interaction between *MUC4* and treatment, with a higher prevalence of ETEC F4 in the SYN group – but only in the *MUC4* non-carrier animals. *MUC4* did not significantly impact the ileal scraping counts at days 4 or 8 PI.

**Figure 4:**
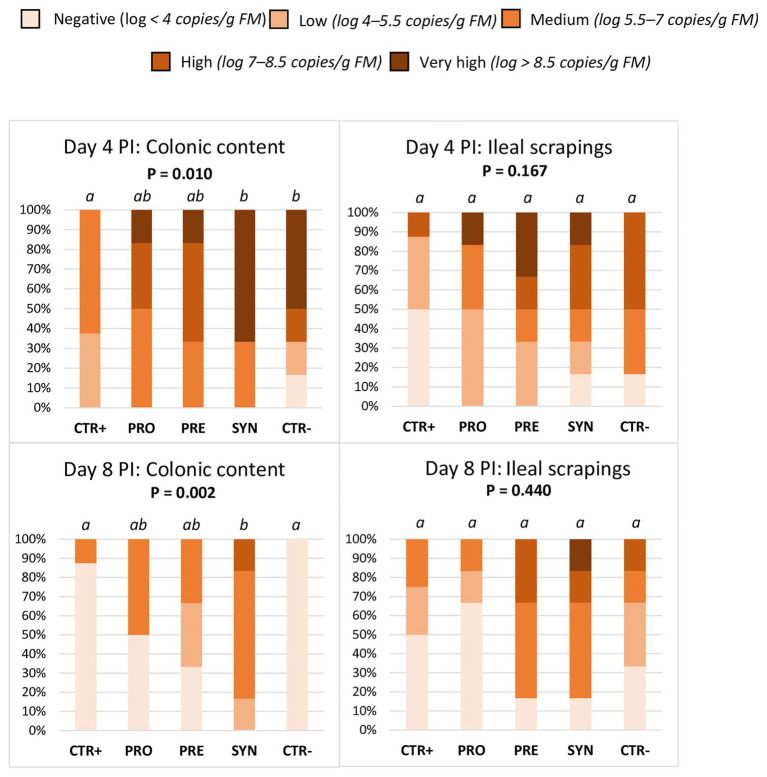
Effect of experimental treatments on the percentage of animals with different levels of ETEC F4 counts in colonic digesta and ileal scrapings on days 4 and 8 post-inoculation (PI). Different animals were sampled on day 4 PI and 8 PI. CTR+: non-inoculated animals receiving placebo; PRO−: inoculated animals receiving the probiotics; PRE: inoculated animals receiving the prebiotic; SYN: inoculated animals receiving the synbiotic; CTR−: inoculated animals receiving placebo. *N* = 6 for all groups except for non-challenged animals (*N* = 8). Values of *p* were obtained using Fisher’s exact test in R software. a and b indicate statistically significant differences between groups. FM, fresh matter; PI, post-inoculation.

When considering all animals, regardless of *MUC4* status (Figure 4), there were differences between treatments in the colonic prevalence of ETEC F4. On day 4 PI, the SYN group showed a higher prevalence compared to the CTR+ and similar to the CTR− group; the PRO and PRE groups showed intermediate levels (*p* = 0.01). On day 8 PI, the SYN group maintained high excretion levels (SYN vs. CTR+; *p* = 0.002), while animals from the CTR− group recovered from the challenge and the values decreased; the PRO and PRE groups showed intermediate values.

### Intestinal Fermentation

Table 5 shows values of intestinal pH, ammonia, lactic acid, and short-chain fatty acid (SCFA) concentrations in ileal and colonic content for the different treatments.

**Table 5:** Effects of experimental treatments on ileal and colonic fermentation.

	Treatment						RSD	*p*
	Day PI	CTR+	PRO	PRE	SYN	CTR−		
**Ileum**								
pH	4	6.49^c^	6.58^bc^	6.94^a^	6.70^abc^	6.86^ab^	*0.169*	<0.001^*^
	8	6.53	6.52	6.41	6.56	6.57	*0.110*	0.044^*^
NH_3_ (mmol/L)	4	1.99	1.86	0.99	1.84	1.53	*0.973*	*0.390*
	8	2.69^a^	1.38^b^	1.52^b^	1.59^b^	2.04^ab^	*0.835*	0.044^*^
Lactic acid (mmol/kg)	4	35.8	18.1	5.6	8.3	16.7	*27.48*	*0.368*
	8	19.8	24.5	27.6	23.1	12.5	*19.60*	*0.742*
SCFA (mmol/kg)	4	3.85	3.59	2.33	4.71	1.99	*2.306*	*0.288*
	8	2.67	3.07	2.59	3.37	3.47	*1.662*	*0.849*
**Colon**								
pH	4	6.04^c^	6.11^bc^	6.35^abc^	6.66^a^	6.54^ab^	*0.389*	0.041^*^
	8	6.02	5.92	6.04	5.95	5.79	*0.307*	*0.149*
NH_3_ (mmol/L)	4	16.4^a^	10.6^ab^	7.2^b^	10.6^ab^	12.7^ab^	*4.86*	0.007^*^
	8	8.84	6.63	7.15	9.02	9.16	*3.01*	*0.477*
Lactic acid (mmol/kg)	4	2.57	0.56	2.71	0.26	0.27	*3.542*	*0.550*
	8	0.85	0.43	0.10	0.00	7.27	*4.877*	*0.119*
SCFA(mmol/kg)	4	124.1	105.7	100.6	75.5	78.1	*33.72*	*0.085*
	8	130.9	136.2	137.3	127.4	145.4	*32.07*	*0.442*
**Molar ratio of SCFA (%)**								
Acetic	4	50.7	58.6	59.6	52.8	55.4	*5.99*	*0.057*
	8	50.3^ab^	55.3^a^	48.7^b^	46.4^b^	48.7^b^	*3.44*	0.004^*^
Propionic	4	26.6	25.1	26.7	28.5	28.1	*3.68*	*0.518*
	8	26.1	25.9	29.7	28.2	25.8	*3.76*	*0.317*
Butyric	4	16.1	12.9	10.2	12.6	11.1	*5.48*	*0.332*
	8	17.6	14.2	14.1	16.7	18.2	*3.33*	*0.118*
Valeric	4	4.20^a^	1.70^b^	1.72^b^	2.30^b^	2.37^ab^	*1.665*	0.047^*^
	8	3.85^ab^	2.97^b^	5.28^ab^	6.23^a^	5.49^ab^	*2.018*	0.011^*^
BCFA	4	2.30^ab^	1.70^b^	1.74^ab^	3.73^a^	2.95^ab^	*1.119*	0.031^*^
	8	2.12	1.65	2.24	2.51	1.77	*0.743*	*0.097*

This table includes values corresponding to pH, ammonia concentration (NH_3_; mmol/L of FM), lactic acid (mmol/L of FM), total short-chain fatty acids (SCFA; mmol/L of FM), and molar ratio of these SFCA. CTR+: non-inoculated animals receiving placebo; PRO: inoculated animals receiving the probiotics; PRE: inoculated animals receiving the prebiotic; SYN: inoculated animals receiving the synbiotic; CTR−: inoculated animals receiving placebo. *N* = 6 for all groups except for non-challenged animals (*N* = 8). Values of *p* were obtained by an ANOVA using the generalized linear procedure in R software. ^a,b,c^Indicate statistically significant differences between groups. BCFA, branched-chain fatty acids; NH_3_, ammonia concentration; PI, post-inoculation; RSD, relative standard deviation; SFCA, short-chain fatty acids. To differentiate RSD and values of p from the results numbers, they are in italics. *indicates that values of p are <0.05 and statistical difference is present.

At the ileal level, the PRE group had the highest pH values at day 4 PI; they were different from the CTR+ and PRO groups (*p* < 0.05), but not from the other challenged groups. At day 8 PI, the PRE group had the lowest pH values (P treatment = 0.044). Ammonia concentrations diminished at day 8 PI in the PRO, PRE, and SYN groups compared to the CTR+ and CTR− groups (*p* = 0.044). There was no treatment effect in lactic acid or total SCFA concentrations.

In the colon, the pH was numerically increased at day 4 PI in all challenged groups, although the differences compared to the CTR+ group were only significant for the SYN and CTR− groups (P treatment = 0.041). The PRE group was the only treatment with reduced ammonia values at day 4 PI compared to the CTR+ group (P treatment = 0.007). There were no differences in lactic acid concentration at any time.

Regarding total SCFA, the SYN and CTR− groups showed a trend for a decreased concentration compared to the other groups (P treatment = 0.085) at day 4 PI but not at day 8 PI. At day 8 PI, there was a marked increase in lactic acid concentration in the CTR− group, although differences with other treatments did not reach statistical significance (*p* = 0.119). The molar ratios of different SCFA showed some changes related to the experimental treatments. On day 4 PI, there was a numerical trend for an increased acetic acid percentage in the challenged groups (P treatment = 0.057), and at day 8 PI, the PRO group showed a higher acetic acid concentration compared to other inoculated piglets (*p* = 0.004). The PRO group showed lower percentages of branched chain fatty acids (BCFA) compared to the SYN group (*p* = 0.049), and valeric acid was reduced in the PRO, PRE, and SYN groups compared to the CTR+ group at day 4 PI (P treatment = 0.047).

**Figure 5:**
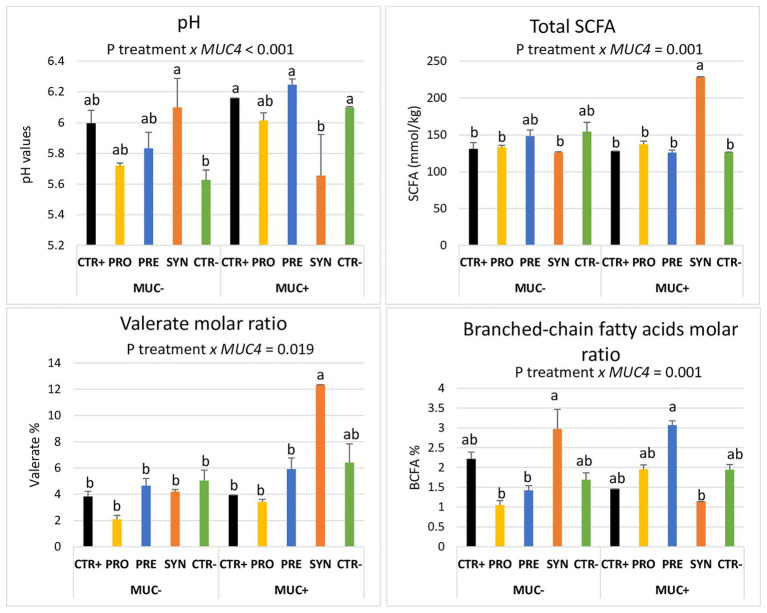
Interactions between *MUC4* gene polymorphism and diets on pH values, total short-chain fatty acids (SCFA), valerate molar ratio, and branched-chain fatty acids molar ratio (BCFA) on day 8 PI. CTR+: non-inoculated animals receiving placebo; PRO−: inoculated animals receiving the probiotics; PRE−: inoculated animals receiving the prebiotic; SYN−: inoculated animals receiving the synbiotic; CTR−: inoculated animals receiving placebo. *N* = 6 for all groups except for non-challenged animals (*N* = 8). *MUC4* represents the effect of polymorphism of the *MUC4* gene. a and b indicate statistically significant differences between groups. Bars correspond to standard error. BCFA, branched-chain fatty acids; *MUC4*+, Mucin 4 susceptible genotype; *MUC4*−, Mucin 4 resistant genotype; SCFA, short-chain fatty acids.

Notably, these treatment-mediated differences with regard to the fermentation profile were influenced by *MUC4* polymorphism when analyzed at day 8 PI. The identified interactions are shown in Figure 5. The SYN group animals showed a lower colonic pH and a higher amount of total SCFA in the colon, but only in the carrier pigs (P interaction < 0.001). Regarding valerate, the SYN group had a sharp increase in the molar ratio, but only in susceptible pigs (P interaction = 0.019). The BCFA molar ratio was increased in resistant animals in the SYN group and in susceptible animals in the PRE group. Finally, piglets with a *MUC4* resistant phenotype showed increased colon ammonia levels on day 4 PI (13.31 vs. 9.79 mmol/L; P *MUC4* = 0.006); there was no interaction with treatments.

### Immune Response

Table 6 presents values corresponding to serum levels of the pro-inflammatory cytokine tumor necrosis factor alpha (TNF-α) and the acute-phase protein pig major acute-phase protein (Pig-MAP).

**Table 6:** Effect of experimental treatments in serum levels of acute-phase protein Pig-MAP and TNF-α.

	Treatment					RSD	*p*
	CTR +	PRO	PRE	SYN	CTR−		
**TNF-α (pg/ml)**							
Day 4 PI	85.7^ab^	97.6^ab^	75.3^b^	118.2^a^	76.7^b^	*23.83*	0.023^*^
Day 8 PI	83.6	74.3	83.1	70.2	93.8	*13.68*	*0.050*
**Pig-MAP (mg/ml)**							
Day 4 PI	0.59^b^	0.76^b^	0.72^b^	1.49^ab^	2.42^a^	*0.986*	0.013^*^
Day 8 PI	0.51^b^	0.57^b^	0.56^b^	2.43^a^	0.62^b^	*0.752*	0.003^*^

CTR+: non-inoculated animals receiving placebo; PRO: inoculated animals receiving the probiotics; PRE: inoculated animals receiving the prebiotic; SYN: inoculated animals receiving the synbiotic; CTR−: inoculated animals receiving placebo. *N* = 6 for all groups except for non-challenged animals (*N* = 8). Values of *p* were obtained by an ANOVA using the generalized linear procedure in R software. ^a,b^Indicate statistically significant differences between groups. TNF-α, tumor necrosis factor alpha; Pig-MAP, pig major acute-phase protein; PI, post-inoculation; RSD, relative standard deviation. To differentiate RSD and values of p from the results numbers, they are in italics. *indicates that values of p are <0.05 and statistical difference is present.

The TNF-α concentration on day 4 PI was higher in the SYN group compared to the CTR− and PRE groups (P treatment = 0.023); any differences did not reach significance with the rest of the groups. At day 8 PI, the SYN group showed the lowest values (*p* = 0.050).

On day 4 PI, the Pig-MAP value was higher in the CTR− compared to the CTR+ group. The PRO and PRE groups had levels closer to the CTR+ group, and the SYN group showed intermediate levels (P treatment = 0.013). On day 8 PI, the Pig-MAP concentration was clearly higher in the SYN group, whereas the rest of challenged groups resembled the CTR+ group level (P treatment = 0.003).

There were no effects due to *MUC4* gene polymorphism.

### Intestinal Histological Structure

The effects of the experimental treatments on villi height, crypt depth, and mitosis number are shown in Table 7.

**Table 7:** Effects of treatments on ileal histomorphological parameters on days 4 and 8 post-inoculation (PI).

	Day PI	Treatment					RSD	*p*
		CTR+	PRO	PRE	SYN	CTR−		
Villi height (μm)	4	311.8^a^	241.4^b^	246.8^b^	245.3^b^	220.9^b^	*34.76*	<0.001^*^
	8	336.8^a^	291.8^ab^	272.1^b^	269.0^b^	266.5^b^	*28.59*	<0.001^*^
Crypth depth (μm)	4	272.4^ab^	271.9^ab^	282.5^a^	251.6^ab^	241.8^b^	*22.19*	0.020^*^
	8	271.3	274.0	271.3	250.5	278.3	*36.17*	*0.710*
Villi height: crypt depth ratio	4	1.14^a^	0.88^b^	0.87^b^	0.97^ab^	0.91^b^	*0.114*	<0.001^*^
	8	1.24^a^	1.06^b^	1.00^b^	1.09^ab^	0.96^b^	*0.099*	<0.001^*^
IEL (cell number/100 μm)	4	0.37	0.50	0.52	0.59	0.38	*0.207*	*0.285*
	8	0.69	0.56	0.66	0.60	0.71	*0.293*	*0.885*
GC (cell number/100 μm)	4	2.38	1.96	2.39	2.95	2.08	*1.031*	*0.513*
	8	1.94	1.77	2.28	2.07	2.76	*0.790*	*0.250*
Mitosis (cell number/100 μm)	4	0.16^a^	0.24^ab^	0.32^b^	0.30^b^	0.31^b^	*0.103*	0.036^*^
	8	0.23	0.22	0.27	0.22	0.26	*0.098*	*0.896*

CTR+: non-inoculated animals receiving placebo; PRO: inoculated animals receiving the probiotics; PRE: inoculated animals receiving the prebiotic; SYN: inoculated animals receiving the synbiotic; CTR−: inoculated animals receiving placebo. *N* = 6 for all groups except for non-challenged animals (*N* = 8). Values of *p* were obtained by an ANOVA using the generalized linear procedure in R software. ^a,b^Indicate statistically significant differences between groups. GC, Goblet cells; IEL, intraepithelial lymphocytes, PI, post-inoculation; RSD, relative standard deviation. To differentiate RSD and values of p from the results numbers, they are in italics. *indicates that values of p are <0.05 and statistical difference is present.

The impact of the ETEC F4 oral challenge was evidenced by the ileal epithelium structure. On day 4 PI, all the challenged groups showed a reduction in the villi height (*p* < 0.001), a phenomenon which was still significant on day 8 PI, except for the PRO group (P treatment < 0.001). When analyzing crypt depth on day 4 PI, the CTR− group had the lowest values, whereas the PRE group had the highest; the difference between the two treatments was statistically significant (*p* = 0.028). According to this result, the villous height:crypt depth ratio was modified by the oral challenge on both sampling days (*p* < 0.001), with reductions in the ratio for all the challenged groups except SYN. In that group, the reductions did not reach statistical significance compared to the CTR+ group (*p* = 0.064).

The challenge was also associated with a higher number of mitotic cells (P treatment = 0.036), although this increase was not significant in the PRO group when compared to the CTR+ group (*p* = 0.649) at day 4 PI. Regarding goblet cells and interepithelial lymphocytes (IELs), there were no significant differences related to the experimental treatments.

*MUC4* significantly affected the number of mitotic cells on day 4 PI: susceptible animals presented reduced mitosis compared to resistant animals (0.23 vs. 0.29 cell number/100 μm, P *MUC4* = 0.049). Furthermore, the villous height:crypt depth ratio on day 8 PI showed an interaction effect (*p* = 0.004). Specifically, the SYN group exhibited a higher villous:crypt ratio compared to the challenged groups, but only in the susceptible animals (1.33, 1.07, 1.01, 1.32, and 0.95 for CTR+, PRO, PRE, SYN and CTR−, respectively).

## Discussion

In this study, a piglet model of ETEC F4 colibacillosis was used to evaluate the efficacy of treatment with *B. longum* subsp. *infantis* CECT 7210 and *L. rhamnosus* HN001, galacto-oligosaccharides, and their combination. The current experimental trial clearly demonstrated the effects caused by the pathogen challenge. Specifically, in all the challenged groups, there was a decrease in feed intake, followed by a reduction in weight gain. Piglets also showed impaired fecal consistency immediately after the inoculation, with evident effects on the intestinal epithelium structure and the Pig-MAP response.

The work also analyses the possible role of the *MUC4* gene in the development of the disease. Actually the presence of ETEC F4-specific receptors on the intestinal brush border of pigs has been associated with *MUC4* polymorphism (Peng et al., 2007; Trevisi et al., 2009) and suggested as criteria for the inclusion of animals in ETEC F4 challenge models (Luise et al., 2019a). As expected, in the present study, animals that were heterozygous or homozygous for the susceptible *MUC4* allele showed the elevated levels of enterobacteria, coliforms, and ETEC F4 on some of the sampling days, as well as a worsened fecal consistency. These results support the usefulness of this candidate gene as marker for the genetic selection of farmed pig toward increased resistance to diarrhea (Jørgensen et al., 2003). However, it is also fair to remark that nor *MUC4* polymorphism or any other candidate marker has yet been confirmed as the univocal causative gene for ETEC F4 susceptibility. Actually Rasschaert et al. (2007) showed that 30% of the genotypic resistant pigs are positive in the *in vitro* villous adhesion assay, suggesting that polymorphisms other than *MUC4* could also been involved. Between those other candidate markers there has been described single nucleotide polymorphisms (SNPs) located on *MUC4*, *MUC13*, *MUC20*, the transferrin receptor (*TFRC*), tyrosine kinase non-receptor 2 (*ACK1*), or the UDP-GlcNAc:betaGalbeta-1,3-N-acetylglucosaminyltransferase 5 (*B3GNT5*) gene (reviewed by Luise et al., 2019a). Moreover, Goetstouwers et al. (2014) found a refined candidate region for F4ab/ac ETEC susceptibility situated proximal to *MUC13* gene, however, it should be noted that this marker maps on a non-coding region. No protocols, in addition to an Illumina chip or a next-generation sequencing (NGS) technique, are available for genotyping the pigs for this marker, making nowadays difficult to routinely use for animal selection. To better dilucidated the role of *MUC4* gen in this study, it had been interesting to determine the phenotypical presence of F4 receptors on brush borders, enterocytes, or villi of the small intestine by *in vitro* test (Van den Broeck et al., 1999). However, as characterization of the F4ab/ac ETEC susceptibility was not initially planned, unfortunately the receptor expression in susceptible pigs could not be confirmed.

Regarding the potential of the probiotic to fight the disease, the probiotic combination of *B. infantis* CECT 7210 and *L. rhamnosus* HN001 ameliorated the impairment in weight gain immediately after ETEC F4 inoculation (day 0–4 PI); the only challenged group that was not significantly different from the CTR+ group. Moreover, in the 4–8 PI period, the PRO group also showed improved weight gain, reaching levels similar to the CTR+ group. This enhanced response could be the result of a competitive exclusion of the pathogen by the probiotics. In fact, at day 4 PI this group showed an ETEC F4 colonic prevalence with values intermediate to those in the CTR+ and CTR− groups. In addition, there was a lower number of fecal enterobacteria and coliform plate counts compared to the CTR− group as well as a reduced number of attached bacteria to the ileal mucus – only the experimental diet group was not different from the CTR+ group. Other authors have reported the beneficial effects of these two probiotic strains against pathogenic agents, albeit when used separately. Barba-Vidal et al. (2017) showed, in a similar piglet model, that *B. longum* subsp. *infantis* CECT 7210 tends to reduce the percentage of animals with countable ileal coliforms and decreases fecal *Salmonella* excretion after an ETEC F4 or a *Salmonella* Typhimurium oral challenge, respectively. Moreover, this strain has antiviral activity when tested against rotavirus in mice (Muñoz et al., 2011) as well as antidiarrhoeal properties in healthy infants (Escribano et al., 2018). Similarly, *L. rhamnosus* HN001 is successful against pathogens such as ETEC and *Staphylococcus aureus* (Gopal et al., 2001; Inturri et al., 2016; Eggers et al., 2018). More recently, in previous studies of our group, we could also demonstrate the positive effects of this probiotic combination against a *Salmonella* challenge, leading to a faster clearance of the pathogen and recovery from the intestinal damage (Rodríguez-Sorrento et al., 2020). This putative reduction in the enteropathogen challenge promoted by the probiotic treatment could have also explained the intermediate level of mitosis observed between the CTR+ and CTR− groups at day 4 PI and the faster recovery of villus height at day 8 PI. These effects could also be associated with the reduced response observed in Pig-MAP at day 4 PI. This treatment showed values between 0.43 and 1.50 mg/ml, which are clearly below the 2 mg/ml considered to be normal in weanlings [Piñeiro et al., 2009; normal (≤1 mg/ml), borderline (1–2 mg/ml) and high levels (>2 mg/ml)]. Pig-MAP is commonly induced by interleukin 6 (IL-6; González-Ramón et al., 2000) that is simultaneously stimulated by nuclear factor kappa B (NF-κB) activation (Brasier, 2010). NF-κB is a protein complex, which controls the expression of genes implicated in inflammation process (Baker et al., 2011). *Bifidobacterium infantis* and *L. rhamnosus* can also modify the expression of IL-6 or NF-kB (Khailova et al., 2014; Gamallat et al., 2016; Ishizuka et al., 2016), an eventuality that is consistent with our results. A possible modulation of the immune response by this probiotic should also be considered. In this regard, previous works with *B. infantis* CECT 7210 have shown consistent increases in ileal IEL (13), and *L. rhamnosus* HN001 is attributed immune-modulating properties (Gill et al., 2001). However, we could not find such an effect in the present study when combined with *L. rhamnosus* HN001.

Galacto-oligosaccharides supplementation alone (the PRE group) in the piglets’ diet also promoted favorable outcomes. Like the probiotics alone, the PRE group presented improved weight gain for the 4–8 PI period, reaching similar values to the CTR+ group (and even numerically higher). Supplementing diets with the GOS alone affected neither enterobacteria nor coliform populations nor ileal histomorphometry, although, like the PRO group, the prevalence of colonic ETEC F4 was between the levels of the CTR+ and CTR− groups. At day 4 PI, the PRE group showed the lowest Pig-MAP level compared to the other challenged groups; only the PRE group was significantly different from the CTR− group and similar to the CTR+ group. In fact, GOS may alleviate inflammation, as shown in several studies (Vulevic et al., 2013; Verheijden et al., 2015). Wang et al. (2019) attributed the modulatory effects of inflammatory state to the ability of GOS to increase anti-inflammatory cytokine IL-10 while decreasing IL-8 by modulating, once again, NF-κB protein complex. Regarding fermentation, the prebiotic encouraged a decrease in colonic and ileal ammonia, data that suggest a possible shift toward a less proteolytic and beneficial microbiota due to its inclusion, as, for example, ammonia may buffer SCFA and block their activity (Davila et al., 2013; Shen et al., 2015).

The interest of mixing the tested probiotic strains with GOS is based on the reported ability of *Bifidobacteria* and *Lactobacillus* to degrade and use GOS as energy source. β-glycosidic linkages connecting saccharides that comprise GOS are hydrolyzed in the colon by these two genera of bacteria that bear β-galactosidases (Andersen et al., 2012; Garrido et al., 2013) and, specifically, *B. infantis* CECT 7210 prefers GOS as a growth substrate rather than other prebiotics (Ruiz et al., 2020). Several works have reported a rise in *Bifidobacteria* and *Lactobacillus* when tested together with different types of GOS, whether or not it is very pure (Hong et al., 2016; Monteagudo-Mera et al., 2016; Kittibunchakul et al., 2018). However, GOS utilization varies depending on the bacterial strain and the oligosaccharide composition (Thongaram et al., 2017). Hence, considering the previously mentioned characteristics, administering these probiotics with GOS potentially would lead to synergistic activity enhancing the benefits that each component produces on its own, however, in the present study, we were not able to demonstrate any additive o synergic effect. In the literature, works evaluating the synbiotic strategy are limited, and the results have not always been consistent. For example, Tanner et al. (2014) and Abrahamse-Berkeveld et al. (2016) observed positive outcomes, including enhanced growth and pathogen inhibition, when mixing a bifidobacteria strain with GOS in both *in vitro* and *in vivo* studies. By contrast, Krumbeck et al. (2018) did not find any synergy in the capacity of improvement intestinal barrier function when combining bifidobacteria and GOS in humans. Previous studies of our group also failed to demonstrate the synergic activities of combination of the strains CECT 7210 and HN001 with FOS in *Samonella* orally challenged piglets (Rodríguez-Sorrento et al., 2020). In the present study, we observed some synergy before the challenge, particularly in the number of enterobacteria and coliforms, which were lower compared to the rest of supplemented groups. This effect could suggest a positive impact of the synbiotic treatment on the resident microbiota, specifically promoting the growth of specific microorganisms, which together with the probiotics, would have displaced enterobacteria. These results are particularly relevant since the 1st week after weaning is one of the most critical periods in the pig’s life, in which they have to cope with numerous stressors and dysbiosis is commonly present (Gresse et al., 2017; Motta et al., 2019). Shifts of microbiota toward bifidobacteria *via* the administration of a synbiotic that contains GOS [±oligofructose (OF)] and strains of *Bifidobacterium* have been reported in trials with healthy newborn babies and infants (Simeoni et al., 2016; Chua et al., 2017). In particular, the latter authors attributed this outcome to the increase of endogenous bifidobacteria and also in piglets by OF (Trevisi et al., 2008; Modesto et al., 2009). However, the outcomes in the SYN group were different after the challenge. During the acute period of the infection (days 0–4 PI), the SYN group showed a larger decrease in feed intake compared to the other treatments, with similar values to challenged pigs that were not supplemented. This feed intake depression was even more pronounced in the 4–8 PI period. According to this reduced feed intake, SYN and CTR− were the only two groups that lost weight in the 0–4 PI period, and the SYN group showed a trend for lower gains compared to the CTR− group in the 4–8 PI period. Together with reduced intake, impaired nutrient utilization associated with diarrhea could also explain weight loss (Clements et al., 2012). Although we were unable to find differences in the fecal score due to the dietary supplementation, the highest fecal scores on day 1 PI occurred in the SYN group, with values that were significantly higher than those found in the PRO group. This trend for a more acute peak of diarrhea in the SYN group is supported by the higher prevalence of ETEC F4 found in the colon on day 8 PI compared to the CTR+ group. This outcome was particularly evident in the groups of animals with the *MUC4* susceptible genotype, consistent with previous findings (Luise et al., 2019b). Moreover, the concentration of inflammatory serum markers was higher in the SYN group. On day 4 PI, the TNF-α value was higher in the SYN group compared to the CTR− group, although it was not different from the PRO or CTR+ groups. Regarding Pig-MAP, whereas on day 4 PI the SYN group showed a level between the CTR+ and CTR− groups, at the end of the trial (day 8 PI), the SYN group maintained markedly high values, in contrast to the rest of the challenged groups that were able to normalize Pig-MAP levels. A higher energy expenditure associated with an inflammatory response could have also contributed to explain the lower performance of the SYN animals.

Based on the above findings, it is evident that the synbiotic combination did not show any additive or beneficial effect on the challenged animals but could even have provided a better opportunity for ETEC F4+ to thrive. Whereas before the inoculation the SYN treatment showed a positive outcome with reductions in the number of enterobacteria and coliforms, after the ETEC challenge the combination of the probiotic and prebiotic could have reduced the ability of the animal to fight the pathogen. An explanation for that phenomenon could be the complex interactions between members of the intestinal microbiota that could have been potentially disturbed by the synbiotic. It is known that a well-established and developed microbiota is characterized by an equilibrium between different microbial groups through cross-feeding, competitive exclusion, and quorum sensing mechanisms that provide to the microbial community with a high resilience against external inputs (Walter et al., 2018). The administration of SYN could have determined changes in the sequence of gut colonization of the young pig during the 1st week post-weaning leading to a microbial ecosystem more susceptible to be colonized by opportunistic pathogens like ETEC F4. The fermentation of GOS by the probiotic along the small intestine, could have increased the amount of probiotic reaching the hindgut and also have reduced the amount of GOS arriving to the colon as intact prebiotic. This hypothesis could explain why the ammonia concentration was only decreased in colon at day 4 PI in the PRE but not in the SYN group as less GOS would have arrived to the colon. Moreover, the reduction observed in enterobacteria and coliforms with SYN, although generally regarded as positive, could have been a consequence of a reduced biodiversity and resilience of the ecosystem. Not necessarily a reduction in number of enterobacteria in the microbial community is always positive. Previous authors have described how commensal Enterobacteriaceae can protect neonates against *Salmonella* colonization through oxygen competition (Litvak and Bäumler, 2019) as well as producing bacteriocins able to eliminate close-related competitors (Stecher, 2015). Seems therefore plausible to hypothesize that the biodiversity of the intestinal community could have been reduced by the synbiotic treatment, making the ecosystem more susceptible to dysbiosis with more niches becoming available for opportunistic/pathogenic bacteria.

Interestingly, the impact of the synbiotic treatment on microbial metabolism seemed to be *MUC4* genotype dependent. The SCFA concentration was increased, and also the molar ratio of valeric acid at day 8 PI, but only in pigs with the *MUC4* susceptible genotype. A more acute course of diarrhea, determined by the susceptible genotype, could have promoted a differential effect on microbial activity, suggesting that the effect of the SYN was somehow challenge dependent. In support of this hypothesis, it is known that the *MUC4* genotype can influence the small intestinal glycomic patterns (Luise et al., 2019b) and the intestinal microbiota (Massacci et al., 2020), facts that could explain the differential response of the susceptible animals to the SYN treatment. The increase observed in the molar proportion of valeric acid with SYN supplementation could be attributed to favored valeric-producing bacteria. In this vain, Van Nevel et al. (2005) observed that the inclusion of galactomannans from locust bean gum increases valeric acid and decreases the mitotic index in crypts, similarly to the present study, where we also found a lower mitosis index and shorter crypts in the susceptible animals of the SYN group.

It is also fair to remark that results from challenge studies like this one are always conditioned by the experimental model of disease and need to be approached with caution. An oral challenge with a single high dose of ETEC F4 is undoubtedly far from what would naturally occur, namely repeated lower doses. Factors, as the inoculated bacterial strains, doses, timing of the inoculum, post-weaning age, and immunity status of the animals, undoubtedly are variables that could determine differential responses (Luise et al., 2019a). One of the factors that could deserve some discussion is timing for inoculation. We inoculated animals 7 days post-weaning (around 28 days of age) aiming to test the potential prophylactic effect of the symbiotic. Inoculating animals in shorter times (i.e., 1–2 days post-weaning) could had better resembled the situation in the farm, tackling the time of the highest risk for intestinal dysbiosis and colonization by ETEC. However, challenging animals only 1–2 days after weaning had limited the possibilities for the symbiotic to exert a prophylactic effect. Considering this, we decided to inoculate animals after 7 days post-weaning, although undoubtedly this could had determined differences with what is found in the field.

Regarding the possible role of the *MUC4* gene on results of this trial, it is known that ETEC attaches to the jejunal brush border through the binding of its fimbriae to many putative receptors. These receptors are carbohydrates of glycoproteins in the intestinal epithelial cells and intestinal mucus, which have been shown to differ among pigs (Van den Broeck et al., 2000; Rasschaert et al., 2007). Different antigenic variants of F4 fimbriae have been identified (Bakker et al., 1991), including the F4ab and F4ac variants, in the strain used in this study. *MUC4* codes for a membrane-bound O-glycoprotein in the mucus layer. A mutation in this gene has been proposed as a useful genetic marker to identify susceptible genotypes (Jørgensen et al., 2003; Luise et al., 2019a). In our study, as expected, *MUC4* susceptible piglets showed a worsened fecal consistency, as well as a lower weight daily gain and higher numbers of enterobacteria, coliforms, and ETEC F4 in colon. Other authors have also described similar associations in challenge and non-challenge conditions (Casini et al., 2003; Trevisi et al., 2015; Luise et al., 2019b; Sterndale et al., 2019); these data support the usefulness of this candidate gene. Nevertheless, not all the evaluated parameters were modified by *MUC4* status. Specifically, neither the number of enterobacteria and coliforms attached to the ileal epithelium nor ileal histomorphology were altered. This outcome could be due to other genes and receptors involved in the susceptibility to ETEC F4 infection (Nguyen et al., 2012). In this regard, Rasschaert et al. (2007) confirmed that the *in vitro* adhesion of F4ac and F4ab ETEC to the villous brush borders is not always associated with the *MUC4* gene, suggesting the existence of at least one other receptor for F4ab/ac fimbria. Indeed, other genes like *MUC13* or *TNRC* (transferrin receptor gene) have also been associated with ETEC F4 susceptibility (Zhang et al., 2008; Jacobsen et al., 2010; Ren et al., 2012).

In our experiment, we did not select animals based on *MUC4* susceptibility, but we determined this characteristic at the end of the trial. Although many studies with ETEC challenges do not apply any control *a priori* or a posteriori on the genetic susceptibility of the animals, we found that this could be of interest to better understand the variability of the data and to elucidate the role of this candidate gene in the piglet response in front of an ETEC F4 challenge. The *MUC4* analysis showed certain unbalanced distribution of susceptible animals between treatments. This could have had an impact on the results, particularly on those parameters that were influenced by *MUC4* polymorphism (performance, fecal consistency, numbers of fecal coliforms, and colonic ETEC F4). When the whole group of animals are considered, the ratio between resistant and susceptible animals varied between 0.7 for SYN to 2.3 for CTR+ (see Table 2). Although it was a reasonable difference, it could partially explain the lower ADFI and ADG observed for the SYN treatment. When contemplating only the sampled animals after euthanasia, the distribution of resistant and susceptible animals was more unbalanced between treatments causing a different scenario between treatments at days 4 and 8 PI. Whereas at day 4 PI, PRO treatment presented a favorable ratio of 5 and SYN of 0.7, at day 8 PI PRO showed a ratio of 0.5 and SYN of 2. Nonetheless, SYN treatment always exhibited higher ETEC F4 counts than PRO, evidencing that, despite the uneven distribution, SYN treatment consistently brought about the same effects both sampling days. Also, it is interesting to remark that in defiance of the association between the *MUC4* polymorphism and fecal counts of coliforms, particularly relevant at day 4 PI (7.43 vs. 6.27 log CFU/g for *MUC4*+ and *MUC4*−, respectively; *p* = 0.027), the SYN treatment was always associated with lower plate counts compared to CTR−, regardless the dissimilar distribution of resistant and susceptible animals (5.90 vs. 8.48 log CFU/g, *p* < 0.01 at day 4 PI and 5.71 vs. 6.62; *p* < 0.001 at day 8 PI). Altogether, it appears that, although the unbalanced distribution of *MUC4* polymorphism could have increased residual variability and have had an impact on the magnitude of the response, the direction and meaning of the observed differences would be consistent.

In conclusion, the multi-strain probiotic *B. longum* subsp. *infantis* CECT 7210 and *L. rhamnosus* HN001 reduced growth impairment after oral ETEC F4 challenge. Furthermore, this supplementation seems to provide competitive exclusion for ETEC F4, because there were fewer colonic enterobacteria and coliforms in the gut and a trend to diminish the pathogen. This outcome could explain the lower Pig-MAP levels and improved villus height found after 1 week of the challenge with this treatment. The supplementation of the diets with galacto-oligosaccharides also diminished the growth impairment induced by the challenge and is associated with lower levels of plasmatic Pig-MAP. This phenomenon suggests a modulation of the inflammatory response by this prebiotic. These beneficial effects were not synergistic when the probiotics and the prebiotic were administered together. The synbiotic treatment had a differential impact on colonic fermentation depending on *MUC4* susceptibility. The differential response in animals with more acute diarrhea suggests that the absence of synergistic effect could be related to the limitations of experimental model that rely on a single exposure to high doses of the pathogen rather than the low-dose exposure that would occur naturally. More research should be performed in this field to understand the complex interactions produced in the gastrointestinal tract, with an emphasis on the microbiota establishment at early ages.

## Data Availability Statement

The original contributions presented in the study are included in the article/supplementary material, further inquiries can be directed to the corresponding author.

## Ethics Statement

The animal study was reviewed and approved by Animal and Human Experimental Ethical Committee of the Autonomous University of Barcelona (Permit No. CEEAH: 4026 DMAH: 10118).

## Author Contributions

AR-S participated in the experimental design and was responsible for the animal trial, laboratory analysis, data analysis, and writing. LC participated in the experimental design, animal trials, data analysis, and writing. PL-C participated in animal trials and data analysis. GC-O, JM-M, and MR-P participated in the experimental design and contributed to data analysis and writing. DL and PT contributed to laboratory analysis, data analysis, and writing. SM-O participated in the experimental design, animal trials, laboratory analysis, data analysis, and writing. All authors contributed to the article and approved the submitted version.

### Conflict of Interest

GC-O, JM-M, and MR-P were employed by the company Laboratorios Ordesa S.L.

The remaining authors declare that the research was conducted in the absence of any commercial or financial relationships that could be construed as a potential conflict of interest.
